# Gestational exposure to phthalates and gender-related play behaviors in 8-year-old children: an observational study

**DOI:** 10.1186/s12940-016-0171-7

**Published:** 2016-08-16

**Authors:** Zana Percy, Yingying Xu, Heidi Sucharew, Jane C. Khoury, Antonia M. Calafat, Joseph M. Braun, Bruce P. Lanphear, Aimin Chen, Kimberly Yolton

**Affiliations:** 1 Cincinnati Children’s Hospital Medical Center, 3333 Burnet Avenue, Cincinnati, OH 45229 USA; 2 Centers for Disease Control and Prevention, 4770 Buford Hwy, MS F-17, Atlanta, GA 30341 USA; 3 Brown University, 69 Brown Street, Providence, RI 02912 USA; 4 Simon Fraser University, 8888 University Drive, Burnaby, BC V5A 1S6 Canada; 5 Department of Environmental Health, University of Cincinnati College of Medicine, 160 Panzeca Way, Cincinnati, OH 45267 USA

**Keywords:** Phthalates, Children, Play, Gender

## Abstract

**Background:**

Phthalates, used in a variety of consumer products, are a group of chemicals that are ubiquitous in the environment, and their metabolites are detectable in most humans. Some phthalates have anti-androgenic properties; a prior study reported an association between gestational exposure to phthalates and reduced masculine behaviors in preschool boys.

**Methods:**

Concentrations of 9 phthalate metabolites were measured in urine collected at 16 and 26 weeks’ gestation from pregnant women enrolled in the HOME Study, a prospective pregnancy and birth cohort. Measures of gender-related play were collected at 8 years of age, including the Gender Identity Questionnaire (GIQ) completed by mothers, and the Playmate and Play Style Preferences Structured Interview (PPPSI) completed by children. We examined these measures as continuous variables using both bivariate and multivariable approaches with adjustment for covariates. Additional analyses included logistic regression of GIQ and PPPSI scores dichotomized by sex at the lower 25th percentile, indicating the least typical behavior.

**Results:**

Mothers’ phthalate metabolite concentrations during pregnancy were similar to the reported national average among US women. All children scored within a typical range on both measures of gender-related play behavior. No statistically significant associations were found between averaged maternal phthalate metabolite concentrations and continuous PPPSI scores or any GIQ scores. For the dichotomized PPPSI; higher maternal monoethyl phthalate (MEP) concentrations were associated with more typical play behaviors for females (O*R* = 0.70, CI = 0.51–0.97). In contrast, higher maternal mono-isobutyl phthalate (MiBP) concentrations were associated with higher odds of membership in the least typical play behaviors group for males (O*R* = 1.69, CI = 1.00–2.86).

**Conclusions:**

In this sample of typically developing children, higher maternal urinary MEP concentrations during pregnancy were associated with more typical gender-related play behaviors in both males and females, and increased urinary MiBP concentrations were associated with less masculine gender-related play behaviors in males.

## Background

Phthalic acid diesters, or phthalates, are a multifunctional group of chemicals found in a variety of consumer products including vinyl flooring, shower curtains, raincoats, bath products, nail polish, perfumes, cosmetics, medications, and food storage containers. Phthalates are ubiquitous in the modern environment and enter our bodies through food consumption, inhalation, or dermal contact [1–3]. After phthalates enter the body, they are quickly metabolized into their respective hydrolytic and oxidative monoester metabolites, conjugated, and excreted into the urine by the kidneys [4]. Di-2-ethylhexyl phthalate (DEHP), one of the most widely used phthalates, is mainly metabolized into mono (2-ethylhexyl) phthalate (MEHP), mono (2-ethyl-5-hydroxyhexyl) phthalate (MEHHP), mono (2-ethyl-5-oxohexyl) phthalate (MEOHP), and mono (2-ethyl-5-carboxypentyl) phthalate (MECPP). Other phthalate metabolites commonly detected in human urine include monoethyl phthalate (MEP) (metabolite of diethyl phthalate), mono-n-butyl phthalate (MnBP) (metabolite of di-n-butyl phthalate), mono-iso-butyl phthalate (MiBP) (metabolite of di-iso-butyl phthalate), monobenzyl phthalate (MBzP) (metabolite of benzylbutyl phthalate), and mono (3-carboxypropyl) phthalate (MCPP) (metabolite of di-n-octyl phthalate and other high molecular weight phthalates).

The long-term consequences of human exposure to phthalates are poorly understood. Of particular concern are the potential effects of gestational exposure to phthalates on infants and children. Studies have shown that phthalates can cross the placenta [5, 6]. Rapid brain development and immature detoxification pathways make fetuses particularly vulnerable to neurotoxicants.

Characterized as endocrine disruptors, there is evidence that some phthalates reduce testosterone levels in both animals and humans [7–11]. Animal studies have shown that gestational exposure to phthalates have a de-masculinizing effect on male genitals [12–15], and studies have also found analogous associations in human males [13, 16]. There is also evidence of sexually dimorphic neurobehavioral changes in rodents exposed prenatally to phthalates [17].

Play behaviors, which are an accepted method to determine gender identity in children, are a critical factor in diagnosing Gender Dysphoria, according to the American Psychiatric Association [18]. In humans, androgens contribute to sexually-dimorphic brain and genital development, especially during late first trimester and early second trimester gestation [19]. Sexually-dimorphic behaviors include toy preference, play style, and visual-spatial abilities [18, 20]. Higher testosterone levels during pregnancy have been associated with more masculine play behaviors in both boys and girls [21], suggesting that hormonal changes in the fetal environment can have effects on brain development. While there is evidence that some phthalates can reduce fetal androgen levels and that fetal androgen levels are related to sexually-dimorphic behaviors, the evidence linking gestational exposure to phthalates with subsequent sexually-dimorphic behaviors is limited. To date, one study reported that maternal urinary concentrations of several phthalate metabolites (MnBP, MiBP, MEOHP, and MEHHP) during pregnancy were associated with less masculine play behaviors among preschool-aged boys, suggesting a possible link between phthalates and gender-related behaviors [22].

Given the potential anti-androgenic action of some phthalates and limited research exploring associations between phthalates and human behavior, the goal of this study was to examine associations of gestational exposure to phthalates with gender-related play behaviors in 8-year old children.

## Methods

### Study participants

From March 2003 to February 2006, we enrolled 468 pregnant women in the Health Outcomes and Measures of the Environment (HOME) Study, a prospective pregnancy and birth cohort study in Cincinnati, Ohio. The HOME Study was designed to investigate associations of exposures to environmental toxicants during gestation and early childhood with subsequent health, growth, and neurobehavioral outcomes. Details of the study have been previously published [23]. Briefly, study eligibility criteria included: ≥18 years old, 16 ± 3 weeks pregnant, living in a home built before 1978 (to address lead exposure aims specific to the primary study), no history of human immunodeficiency virus, and not taking any anti-seizure or thyroid medication. A total of 398 women stayed in the study and delivered 407 infants. Children have attended visits in our study clinic annually from 1 to 5 years of age and again at 8 years of age. The current analysis focused on 227 children who completed the 8-year visit and had both maternal biological samples from gestation, gender-related play measures from the 8-year visit, and relevant covariates available. The Institutional Review Boards at Cincinnati Children’s Hospital Medical Center, participating delivery hospitals, and the Centers for Disease Control and Prevention (CDC) approved the study procedures, and written, informed consent was obtained from all women for participation of themselves and their children.

### Urinary phthalate metabolites measurement

At approximately 16 and 26 weeks, enrolled mothers provided urine samples in polypropylene specimen cups that were lot tested and certified to be phthalate free. The samples were processed and stored at or below −20 °C until analysis at the CDC Environmental Health Laboratories, using procedures described elsewhere [24], to quantitate the concentrations of 9 phthalate metabolites: MEP, MnBP, MiBP, MBzP, MCPP, MECPP, MEHHP, MEOHP, and MEHP.

### Gender identity questionnaire

The Gender Identity Questionnaire (GIQ) [25] is a parent-completed survey designed to assess children’s variance from normative gendered behaviors associated with their biological sex. It consists of 16 questions relating to a variety of sex-typed behaviors, and versions specific for boys and girls were used. Two questions addressing romantic interests and a bipolar masculinity-femininity rating were not included in our questionnaire in accordance with previous research that omitted these items [26]. Caregivers who completed the 8-year study visit (98 % of respondents were mothers) selected the frequency with which their child participated in each specified activity. Selections were scored on a scale of 1–5, where 1 was the most sex-atypical choice and 5 was the most sex-typical choice. The mean of the individual responses to each question was used for analysis. Questions answered “not applicable” were not included in the mean score, with 3 questions having a “not applicable” option. A higher final mean score indicated more gender-typical play behaviors.

### Playmate and play style preferences structured interview

The Playmate and Play Style Preferences Structured Interview (PPPSI) [27] is validated as a clinical tool administered directly to the child by a trained research assistant in a clinic setting. This instrument is designed to assess children’s gender-related play preferences. It consists of 40 pairs of drawings shown to children in addition to standardized, scripted verbal cues asking the child to choose a preferred stick-figure playmate between 2 pictured scenarios. Twelve “playstyle” choices depict 2 non-gendered figures, 1 engaged in a masculine-type activity, and 1 engaged in a feminine-type activity. Fourteen “playmate” choices depict a boy and a girl shown with gender-concordant play activities. The twelve “conflict” choices ask the child to choose between a girl engaged in a masculine activity or a boy engaged in a feminine activity. The final 2 “social group” choices show a target playmate engaged with different peer groups. For the “playstyle” and “playmate” items, every masculine choice was coded as zero, and every feminine choice was coded as 1. For the “conflict” items, the choice of a boy engaged in a feminine activity was coded as 1, giving an absolute range of 0–40 for each child, with higher scores suggesting more feminine play preferences. To facilitate comparisons between the GIQ and PPPSI within our sample, we rescored the responses from boys so that a masculine choice was a 1, and a feminine choice was a zero. Thus, higher PPPSI scores represent sex-typical behavior and the lower scores represent less sex-typical behavior, similar to the scores of the GIQ.

Both the GIQ and PPPSI can differentiate children with clinically diagnosed Gender Identity Disorder from controls. Use of ≤3.54 as a cut-point for the GIQ yields sensitivity of 87 % and specificity of 95 %; no similar cut-off values are available for interpretation of the PPPSI for estimating sensitivity and specificity within a gender-typical sample [26, 28]. Additionally, both measures have been validated in several age groups, including age 8. We are unaware of any publications predicting future gendered behaviors from either the GIQ or PPPSI.

### Statistical analysis

Statistical Analysis System (SAS®) version 9.3 was used for all analyses [29]. Initially, the data were examined for missing values, distributional properties, and outliers. For phthalate metabolite concentrations below the limit of detection (LOD), we replaced values with the LOD/(square root of 2), as is common in research on environmental toxicant exposures [30]. We applied creatinine-normalization by dividing the phthalate metabolite concentrations by the measured urine creatinine concentration [31]. We then log_2_-transformed the creatinine-normalized phthalate concentrations to reduce the influence of extreme values and averaged values from the 16 and 26 week concentrations - referred to as mean gestational phthalate concentrations hereafter. Values from the 2 time points were averaged to account for the temporal variability of urinary phthalate metabolite concentrations in pregnant women and to gain a perspective of overall exposure throughout gestation [32]. We set a significance level of *p* = 0.05 for all analyses.

Demographic variables included race, sex, and socioeconomic status (household income and mother’s education) measured at the 8-year visit. Older siblings’ sex has been shown to influence play behaviors of younger siblings [33] and was utilized as a covariate, defined as being the same, different, or both compared to the target child. The Parenting Relationship Questionnaire (PRQ) is a standardized, parent-completed measure of the parent-child relationship with subscales measuring attachment, communication, discipline practices, involvement, parenting confidence, satisfaction with school, and relational frustration [34]. This measure was completed by caregivers at the 8-year visit.

Due to known anti-androgenic properties in animals and previous human research suggesting sexually dimorphic responses to certain phthalates [35], we a priori determined to conduct sex-stratified analyses on all collected data. In bivariate analyses, we tested associations between the independent variables (gestational mean phthalate metabolites concentrations), the dependent variables (GIQ and PPPSI total scores, both as continuous variables), and the potential covariates using Pearson correlation, and utilized analysis of variance (ANOVA) as appropriate. We then conducted multivariable analyses, adjusting for the potential covariates indicated above. Backward elimination of variables was used to determine the most parsimonious multivariable models. A priori, we decided to retain child race in all models, regardless of statistical significance, as this variable has been influential in other analyses of infant and child behavior within this cohort [36–38]. Other covariates were retained in the final models if they were statistically significant (*p* < 0.05) or if the beta coefficient for the independent variable of interest was changed by more than 10 % by removal of the covariate from the full model. Final multivariable models for GIQ included child race, mother’s education, and PRQ Relational Frustration subscale t-score as covariates; and final multivariable models of PPPSI included child race and mother’s education as covariates.

Given our non-clinical typically developing sample, we anticipated that atypical play behaviors would be uncommon in the cohort. We therefore conducted secondary analyses for both GIQ and PPPSI, where scores were examined as binary outcomes (in the lowest quartile of scores vs. all others), to maximize our ability to identify even slight variations from typical patterns of behavior. For convenience purposes, we hereafter refer to the group scored in the lowest quartile as the least typical behaviors group. We additionally conducted a tertile-based sensitivity analysis in which the least typical behaviors group was defined as scoring in the lowest tertile. We calculated a DEHP summary measure as the molar sum of urinary concentrations of MECPP, MEOHP, MEOHP, and MEHP. Finally, we examined the association between an anti-androgenic summary measure and the gender-related play behavior outcomes. The anti-androgenic summary measure was calculated as the sum of urinary molar concentrations of the following metabolites of phthalate exposures shown to have anti-androgenic effects: MECPP, MEHHP, MEOHP, MEHP, MiBP, MnBP, and MBzP.

## Results

Our final sample included 227 of 240 (94.6 %) children whose mothers provided a urine sample during pregnancy and who completed the 8-year clinic visit yielding both the GIQ and PPPSI. At delivery, mothers were on average 29 years of age, and 66 % were married (Table 1). Urine samples were provided by 90 % of mothers at both 16 and 26 weeks gestation; 8 % only provided a sample at 16 weeks, and 2 % only provided a sample at 26 weeks. Children were on average 8.1 years at the visit, 45 % male, and 59 % white.

**Table 1:** Maternal and Child Characteristics (*n* = 227)

	Frequency (SD)	%
Maternal Characteristics		
Age at delivery in years^a^	29 (6)	
Marital Status at delivery		
Married	146	66
Not married, living with partner	31	14
Not married, living alone	57	20
Household income in K$^b^	75 (27.5–105)	
Education		
< = HS or GED	37	16
Some college or college graduate	140	62
Graduate or professional school	48	21
Relational Frustration T-score^a^	47	10
Child Characteristics		
Male	101	45
Race		
White, non-Hispanic	130	57
Black, non-Hispanic	82	36
Other	15	7
Age in years^a^	8.2 (0.6)	
Older Siblings		
None	111	49
Same sex only	38	17
Opposite sex only	47	21
Both male and female	28	13

^a^Mean (SD)

^b^Median (25th-75th percentile)

Maternal urinary phthalate concentrations were generally comparable to national levels [1]. However, MEHP concentrations were twice the nationally reported levels at both time points (Table 2). The Pearson correlations between 16 week and 26 week concentrations among the 9 phthalate metabolites ranged from *r* = 0.03 to *r* = 0.48. Neither measure of gender-related play behaviors showed evidence of deviation from normality. No children in this sample had scores on the GIQ or PPPSI that conferred clinically relevant scores indicating gender dysphoria. Correlation between the GIQ and PPPSI was weak, but positive (Pearson *r* =0.11, *p* = 0.12).

**Table 2:** Geometric mean urinary phthalate metabolite concentrations in HOME Study pregnant women and US women

Metabolite (ng/mL)	LOD (ng/mL)	U.S. Females 2003–04 (*n* = 1355)		HOME Study 16-week (*n* = 226)			HOME Study 26-week (*n* = 218)		
		GM	95 % CI	GM	95 % CI	% > LOD	GM	95 % CI	% > LOD
∑DEHP^a^		*Not available		87.9	73.4–105.3	100	65.9	55.2–78.5	100
MEHP	1.2	2.15	1.92–2.42	4.9	4.1–6	77.4	4.3	3.6–5	78
MEHHP	0.7	19.7	17.4–22.2	27	22.3–32.7	99.6	19.4	16.1–23.4	99.1
MEOHP	0.7	13.4	11.9–15.1	20.1	16.7–24.2	100	15.9	13.2–19.2	98.6
MECPP	0.6	31.9	28.1–36.2	39.3	33–46.9	100	29.1	24.5–34.6	100
MCPP	0.2	2.63	2.44–2.83	2.4	2.1–2.8	98.2	1.6	1.4–1.9	96.3
MBzP	0.3	9.33	8.53–10.2	9.3	7.7–11.3	98.7	7.7	6.2–9.4	98.2
MiBP	0.3	3.56	3.19–3.97	4.8	4.1–5.7	96	3.7	3.1–4.4	94.5
MnBP	0.6	22.2	21.2–23.3	24	20.7–27.7	100	20.6	17.3–24.5	100
MEP	0.8	125	106–148	141.8	118–170.4	100	115.3	92.7–143.3	100
Creatinine^b^ (mg/dL)	5	119	112.6–125.0	132	(89)	100	109	(82)	100

^*^No National levels available for di (2-ethylhexyl) phthalate (DEHP) summary measure (∑DEHP)

^a^∑DEHP expressed as MEHP, ng/mL (= molar sum of (MECPP, MEOHP, MEOHP, MEHP) in nmol/L *278/1000)

^b^Mean (SD) presented to allow comparison with available normative data [45]

**Fig. 1:**
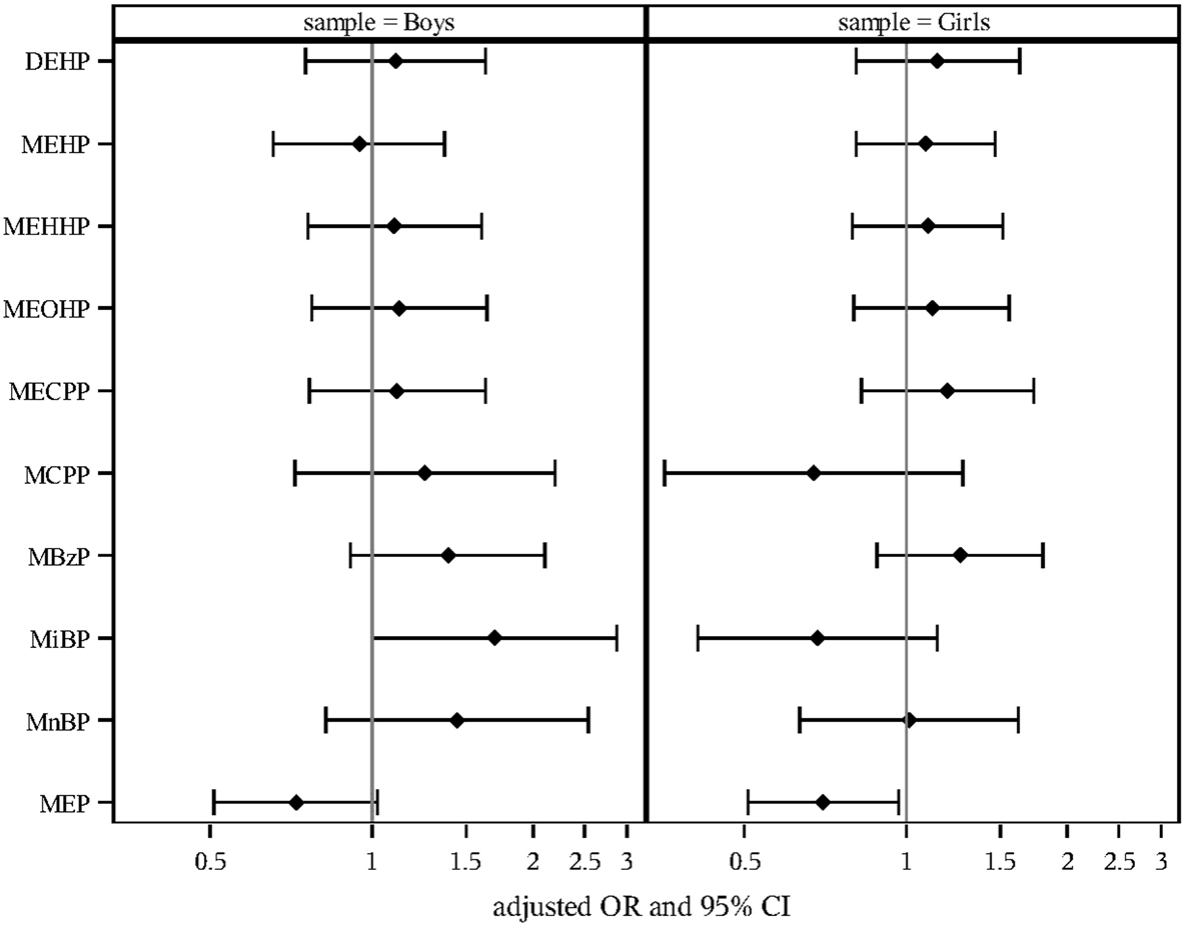
Adjusted Odds Ratio of Low PPPSI Score with Doubling of Maternal Urinary Phthalate Metabolite Concentration

In bivariate analyses stratified by sex, we found no significant associations between phthalate metabolite concentrations and the GIQ or PPPSI scales examined as continuous variables. With adjustment for covariates, we observed no significant associations between urinary phthalate metabolite concentrations and the 2 outcome measures (Tables 3 and 4). Secondary analyses of the GIQ revealed no significant associations between phthalate metabolites concentrations and the odds of being in the least typical behaviors group (Table 3). However, in secondary analyses of the PPPSI, we found higher maternal MEP concentrations were associated with lower odds of membership in the least typical behaviors group for females (O*R* = 0.701, CI = 0.51–0.97, *p* = 0.031), and as a non-significant trend for males (O*R* = 0.721, CI = 0.51–1.02, *p* = 0.07). In contrast, higher maternal MiBP concentrations were associated with higher odds of membership in the least typical behaviors group for males only (O*R* = 1.69, CI = 1.00–2.86, *p* = 0.049 (Table 4, Fig. 1). The sensitivity analysis where the least typical behaviors group was defined as scoring in the lowest tertile yielded no significant results (results not shown). We found no significant associations with the DEHP summary (Tables 3 and 4) or the anti-androgenic summary measure (results not shown).

**Table 3:** Adjusted Associations of Gestational Urinary Phthalate Metabolite Concentrations with the GIQ

Metabolites	GIQ Total Score (Continuous Distribution)						Low GIQ score (GIQ Dichotomized at the < 25th percentile)^b^					
	Males			Females			Males			Females		
	Estimate	95 % CI	*p*	Estimate	95 % CI	*p*	OR	95 % CI	*p*	OR	95 % CI	*p*
∑DEHP^a^	0.01	−0.03, 0.06	0.52	0.03	−0.02, 0.07	0.21	1.13	0.77–1.68	0.53	0.93	0.68–1.27	0.64
MEHP	0.01	−0.02, 0.04	0.61	0.02	−0.01, 0.06	0.21	1.22	0.86–1.73	0.27	0.96	0.71–1.21	0.57
MEHHP	0.01	−0.02, 0.04	0.55	0.03	−0.01, 0.06	0.21	1.15	0.79–1.68	0.47	0.92	0.69–1.22	0.56
MEOHP	0.01	−0.02, 0.04	0.56	0.03	−0.01, 0.07	0.16	1.16	0.79–1.69	0.46	0.91	0.71–1.30	0.51
MECPP	0.01	−0.02, 0.04	0.44	0.03	−0.02, 0.07	0.28	1.09	0.73–1.62	0.68	0.95	0.69–1.31	0.77
MCPP	0.02	−0.03, 0.07	0.43	0.01	−0.07, 0.08	0.93	1.15	0.66–2.00	0.62	1.08	0.63–1.87	0.78
MBzP	−0.01	−0.04, 0.02	0.59	−0.01	−0.05, 0.03	0.67	0.97	0.65–1.46	0.36	0.93	0.71–1.21	0.8
MiBP	0.01	−0.03, 0.05	0.58	0.01	−0.05, 0.07	0.76	1.25	0.78–1.99	0.36	0.81	0.54–1.22	0.32
MnBP	0.02	−0.03, 0.06	0.53	−0.01	−0.06, 0.05	0.84	1.32	0.76–2.29	0.32	1.17	0.78–1.75	0.46
MEP	−0.01	−0.03, 0.02	0.64	0.01	−0.02, 0.05	0.47	1.18	0.87–1.61	0.29	0.92	0.70–1.12	0.53

All values are based on a log_2_-transformed exposure and adjusted for race, mother’s education, and relational frustration score. Metabolite concentrations are the mean of the 16 and 26 week gestational urine samples and corrected for creatinine

^a^∑DEHP expressed as MEHP, ng/mL (= molar sum of (MECPP, MEOHP, MEOHP, MEHP) in nmol/L *278/1000)

^b^Dependent variable is a binary indicator of low GIQ score defined as GIQ score <25th percentile of the GIQ score distribution

**Table 4:** Adjusted Associations of Gestational Urinary Phthalate Metabolite Concentrations with the PPPSI

Metabolites	Continuous Distribution						Dichotomized Distribution (<25th percentile vs. all others)^b^					
	Males			Females			Males			Females		
	Estimate	95 % CI	*p*	Estimate	95 % CI	*p*	OR	95 % CI	*p*	OR	95 % CI	*p*
∑DEHP^a^	−0.41	−1.12, 0.49	0.49	0.19	−0.80, 0.70	0.92	1.11	0.75–1.63	0.60	1.15	0.81–1.63	0.45
MEHP	−0.07	−0.8, 0.66	0.85	−0.03	−0.68, 0.62	0.93	0.95	0.66–1.37	0.77	1.09	0.81–1.47	0.58
MEHHP	−0.23	−1.00, 0.54	0.56	0.03	−0.67, 0.74	0.93	1.10	0.76–1.60	0.61	1.10	0.79–1.52	0.57
MEOHP	−0.28	−1.06, 0.5	0.48	0.02	−0.71, 0.74	0.96	1.13	0.77–1.654	0.54	1.12	0.80–1.56	0.52
MECPP	−0.37	−1.17, 0.43	0.37	−0.10	−0.90, 0.70	0.81	1.11	0.72–1.63	0.58	1.20	0.82–1.74	0.35
MCPP	−0.65	−1.81, 0.52	0.28	0.35	−0.98, 1.69	0.60	1.26	0.72–2.20	0.43	0.67	0.35–1.28	0.23
MBzP	−0.39	−1.21, 0.43	0.36	−0.22	−0.97, 0.52	0.56	1.39	0.91–2.11	0.13	1.26	0.88–1.81	0.20
MiBP	−0.55	−1.50, 0.41	0.27	0.15	−0.90, 1.21	0.78	**1.69**	**1.00**–**2.86**	**0.049**	0.68	0.41–1.14	0.15
MnBP	−0.41	−1.53, 0.71	0.49	0.19	−0.83, 1.20	0.72	1.44	0.82–2.53	0.21	1.01	0.63–1.62	0.96
MEP	0.28	−0.34, 0.9	0.38	0.55	−0.08, 1.18	0.09	0.72	0.51–1.02	0.07	**0.70**	**0.51**–**0.970**	**0.03**

All values are for each doubling of exposure, and adjusted for race and mother’s education. Bolded values are statistically significant at *p* <.05. Metabolite concentrations are the mean of the 16 and 26 week gestational urine samples and corrected for creatinine

^a^∑DEHP expressed as MEHP, ng/mL (= molar sum of (MECPP, MEOHP, MEOHP, MEHP) in nmol/L *278/1000)

^b^<25th percentile group has the least typical play behavior

## Discussion

In this study of typically developing children from a cohort that has been followed since the second trimester of gestation, we found no significant associations between maternal phthalate concentrations during pregnancy and gender-related play behaviors in children at age 8 years when the play measures were examined as continuous variables. In contrast, when child-reported play preferences acquired with the PPPSI were dichotomized, maternal concentrations of some phthalate metabolites during pregnancy were associated with play behaviors at 8 years. Specifically, higher MEP concentrations were associated with more concordant gender-related play behaviors in girls, and MiBP concentrations were associated with less masculine play behavior in boys. No significant findings were revealed with respect to the parent-reported GIQ.

The associations found between MEP, MiBP, and gendered-related play preferences are unique in several ways. Our findings suggest that individual metabolites may act in opposite direction to one another, with MEP associated with more sex-typical play behaviors in females, and MiBP associated with less sex-typical play behaviors in males. The MiBP finding is consistent with the results reported by Swan [22] in which boys whose mothers had higher concentrations of this metabolite during pregnancy exhibited less masculine play behaviors. However, our lack of significant findings with regard to MnBP, MEOHP, and MEHHP are not consistent with those of Swan.

In humans, androgen levels in both males and females are an important predictor of sexually dimorphic behaviors [39]. Gender, which is a fluid and continuous characteristic determined by an individual’s perception of oneself, should not be confused with biological sex, which is determined by chromosomes [40]. Although the terms “gender” and “biological sex” are often considered interchangeable, experts consider gender to exist on a spectrum that can vary along with or in contrast to an individual’s biological sex. Studying school-age children provides more reliable information than that gathered from younger children because gendered patterns of behavior stabilize in the school-age years [41, 42].

It is important to note that none of the children who participated in this study had scores on the gender-related play measures that would indicate gender dysphoria. The cohort fell firmly within accepted score ranges for children used as controls in studies validating both measures [26, 28]. Our data suggest that gestational exposure to diethyl phthalate (DEP) and di-isobutyl phthalate (DiBP), the precursors of MEP and MiBP, respectively, may be associated with subtle shifts on the gender spectrum that are statistically significant for children <25th percentile, yet still within typical ranges for the sexes.

Although most human phthalate exposure studies have reported associations with behavioral and physical health outcomes for males, a limited number have reported effects in females. In females, these limited findings have been related particularly to body feminization and thelarche during puberty and have not been associated with play behaviors [22, 43]. Our results, suggestive of increases in both expected and unexpected play behaviors with exposures to certain phthalates in males and females, provide novel evidence that typical levels of such exposures may have subtle, yet measurable, neurobehavioral effects in humans.

A lack of significant associations between MEP or MiBP concentrations and the GIQ may be related to the fact that it was a parent-report measure, while the PPPSI was a self-report measure. While both the GIQ and PPPSI measure gender-related play behaviors, our results indicate a non-significant correlation between the 2 measures. This is consistent with the literature indicating parent and child reports on child behavior are poorly correlated and that child perspectives on complex behaviors should be used preferentially [44].

Although the exact mechanism underlying the anti-androgenic actions of phthalates is still poorly understood, there is evidence linking phthalate exposure to androgen synthesis. Phthalates have been shown to decrease synthesis of sex steroids, and are thought to interfere with steroid trafficking and synthesis in the testis [45]. In addition, phthalates may interfere with aromatase activity [46], which plays a major role in the conversion of testosterone to estradiol, and may therefore be important for brain masculinization [19]. Further, the effects of phthalates on aromatase have been shown to be sex-differentiated in animals [47].

Although this study has a modest sample size of 227 children, we collected exposure measures twice during gestation and 2 different measures of gender-related play behaviors at age 8 years. Our 2 gestational exposure samples, collected at 16 and 26 weeks, cover much of the fetal period of brain development and masculinization, which begins around 15 weeks gestation [19]. However, because of the complexity of proposed phthalate toxicity mechanisms, we cannot be sure that we have accurately assessed exposure during the most sensitive windows of development. In addition, urinary phthalate concentrations exhibit moderate to substantial within-person variation, which could result in non-differential exposure misclassification and attenuated point estimates [48]. While many covariates and confounders were explored in multivariable models, it is possible that there are unmeasured confounders of which we are not aware. Additionally, the potential role of co-exposure to other endocrine disrupting chemicals was not explored in this study but should be addressed in future work. Finally, the lack of significance in our tertile-based sensitivity analysis suggests that our results may be sensitive to a threshold value. Future research is needed to establish meaningful cut points for the GIQ and PPPSI in populations of typically-developing children.

This study illustrates the importance of identifying neurobehavioral measures that can be used in typically developing children to detect fine gradations in gendered behavior. Currently existing neurobehavioral measures for sexually dimorphic behavior are designed to aid in the diagnosis of Gender Dysphoria. Therefore, presently available measures of play-behaviors are sub-optimal for use in studies such as this one, where the effects of exposure may have more subtle implications on behavior.

## Conclusions

In this cohort, dichotomized analyses examining the least typically behaving children in comparison with remaining cohort members showed that gestational DEP exposure was associated with more expected gender-related play behaviors in females, and gestational DiBP exposure was associated with less masculine play behaviors in males. We found no significant relationships between other measured phthalates and play behavior. Additionally, in continuous models, we found no association between gestational exposure to phthalates and gender-related play behaviors. As exposure to phthalates is prevalent in the general population, it is important to understand the potential role of such exposures in sexually-dimorphic behaviors.

## Abbreviations

ANOVA: Analysis of Variance
CDC: Centers for Disease Control and Prevention
DEHP: Di-2-ethylhexyl phthalate
DEP: Di-ethyl Phthalate
DiBP: Di-isobutyl Phthalate
GIQ: Gender Identity Questionnaire
HOME Study: Health Outcomes and Measures of the Environment Study
LOD: Limit Of Detection
MBzP: Monobenzyl Phthalate
MCPP: Mono (3-carboxypropyl) Phthalate
MECPP: Mono (2-ethyl-5-carboxypentyl) Phthalate
MEHHP: Mono (2-ethyl-5-hydroxyhexyl) Phthalate
MEHP: Mono (2-ethylhexyl) Phthalate
MEOHP: Mono (2-ethyl-5-oxohexyl) Phthalate
MEP: Monoethyl Phthalate
MiBP: Mono-iso-butyl phthalate
MnBP: Mono-n-butyl Phthalate
PPPSI: Playmate and Play Style Preferences Structured Interview
PRQ: Parenting Relationship Questionnaire
SAS: Statistical Analysis System
